# Effects of anthropogenic influences in the DNA methylation and expression of genes involved in the metabolism of *Geophagus surinamensis* from the Pará River (Amazon, Brazil)

**DOI:** 10.1007/s10646-026-03093-w

**Published:** 2026-06-01

**Authors:** Luana Beatriz Sales Pinon, Flávia dos Santos Tavares, Bruno Rafael Ribeiro de Almeida, Cesar Martins, Luis Adriano Santos do Nascimento, Adauto Lima Cardoso, Renata Coelho Rodrigues Noronha

**Affiliations:** 1 Laboratory of Genetics and Cell Biology, Center for Advanced Biodiversity Studies, Institute of Biological Sciences, Federal University of Pará, Belém, 66075-750 PA Brazil; 2 State University of Pará (UEPA), Cametá, 68400-000 PA Brazil; 3 Integrative Genomics Laboratory, Institute of Biosciences, São Paulo State University, Botucatu, 18610-307 SP Brazil; 4 Oils Laboratory, Institute of Biological Sciences, Federal University of Pará, Belém, 66075-750 PA Brazil

**Keywords:** epigenetics, methylation, xenobiotics, *cyp1b1*, *slc16a12b*

## Abstract

The Amazon region has been experiencing increasing anthropogenic pressures, especially in Barcarena, Pará, Brazil, where industrial activities such as aluminum refining and chemical processing have led to repeated contamination events. This study aimed to understand how multiple environmental stressors from an industrial complex affect *Geophagus surinamensis*, a native Amazonian fish, at the molecular level. We analyzed muscle, liver, and gill tissues of *G. surinamensis* collected in Barcarena, Abaetetuba, and Breves. We conducted DNA methylation analysis of the promoter regions of the *cyp1b1* (involved in pollutant metabolism) and *slc16a12b* (a molecule transporter) genes using methylation-specific PCR (MSP) and quantified their expression. A hemimethylation pattern was found in all studied tissues and sampling sites for the *cyp1b1* gene, whereas methylation patterns were more frequently observed for *slc16a12b*. Gene expression varied across sites and tissues. Muscle showed the highest expression of both genes, followed by liver and gills, with *cyp1b1* more active than *slc16a12b*. In the gills, the expression levels of the two genes were positively correlated. These findings show that *G. surinamensis* exhibits biological responses to pollution and can be used as a bioindicator species. The study highlights the importance of using native species and molecular biomarkers to monitor the environmental impacts of industrial pollution in the Amazon.

**Supplementary Information:**

The online version contains supplementary material available at 10.1007/s10646-026-03093-w.

## Introduction

The species *Geophagus surinamensis*, commonly referred to as acará-tinga, is endemic to South America and widely distributed throughout the Amazon Basin (Hauser and Lopez-Fernandez 2013). It holds considerable socio-environmental relevance, particularly among riverine communities, where it represents an important food resource and supports subsistence fisheries (Sousa et al. 2020). Ecologically, this species plays a significant role in Amazonian freshwater ecosystems, exhibiting territorial behavior (Lowe-McConnell 1969, 1991) and defending specific areas for feeding and reproduction (Kullander 2003), thereby contributing to trophic organization and faunal structuring (Darwall et al. 2018). In addition, species of the genus *Geophagus* are characterized by a benthic substrate-sifting feeding strategy, involving the ingestion and processing of bottom sediments in search of invertebrates and organic matter (Kullander 1986). Because aquatic sediments function as reservoirs for heavy metals and other persistent pollutants, this ecological trait may increase exposure to sediment-associated contaminants and favor their bioaccumulation in internal organs. Owing to its ecological abundance, trophic importance, and sediment-associated feeding behavior, *G. surinamensis* constitutes a relevant biological model for investigating contaminant accumulation and associated molecular responses in Amazonian freshwater environments.

In this context, the amazon basin stands out as the largest tropical freshwater network on the planet (Amin 2015), encompassing an area exceeding six million square kilometers (Salati and Vose 1984; Venticinque et al. 2016; Latrubesse et al. 2017). This extensive hydrographic system harbors one of the greatest known diversities of freshwater fish species and supports essential ecosystem services, including nutrient cycling, fishing activities, and the provision of drinking water for millions of people (Blackman et al. 2021). Given its biological and functional richness, the abundance of aquatic resources makes the amazon basin a strategic focal point of global interest for environmental conservation efforts.

Nevertheless, the Amazon Basin is increasingly exposed to a range of anthropogenic stressors, including deforestation, hydroelectric dam construction, mining, agriculture, and contamination by microplastics and metals (Dudgeon et al. 2006; Pelicice et al. 2015 Jézéquel et al. 2020; Leal et al. 2020). As such, the degradation of the Amazon’s freshwater ecosystems has far-reaching consequences that extend beyond environmental dimensions, affecting biological (Pringle 2001; Cantera et al. 2022), economic, and social systems (Dolbec et al. 2001). Biologically, freshwater fish, which act as key regulators of trophic dynamics and nutrient cycles, are experiencing population declines and shifts in species composition as a result of habitat alteration and contaminant exposure (Barlow et al. 2018; Darwall et al. 2018). Economically, the decline of fish stocks threatens food security and household incomes in riverine communities that depend on artisanal fisheries (Dolbec et al. 2001). Socially, the bioaccumulation of toxic substances such as microplastics and metals in fish tissues poses significant public health risks, particularly in regions where fish constitute a dietary staple (Oliveira et al. 2010; Vieira et al. 2013; Faial et al. 2015).

The Barcarena Industrial Complex, located in the state of Pará, serves as a prominent example of the environmental effects associated with industrialization in the Amazon region (De Farias 2023). This major industrial hub hosts the largest aluminum refinery in Latin America, as well as production units for kaolin, fertilizers, and chemical industries (Coutinho et al. 2023). The expansion of this industrial center into riverine areas has led to the discharge of industrial effluents and increased deforestation, thereby elevating pollution levels in local rivers and streams (Morales et al. 2025). The effects of this contamination include the presence of metals such as aluminum, mercury, and cadmium in water bodies, compromising environmental quality and directly affecting aquatic ecosystems (Lemos and Pimentel 2020). Analyses of metal concentrations in water and sediment samples from the municipalities of Abaetetuba, Barcarena, and Breves have been previously conducted and published (Marcelino et al. 2025), providing environmental baseline data for these sampling locations.

Beyond ecological consequences, these impacts extend to human populations. Riverside communities near the Barcarena Complex, which rely on river water and fishing for sustenance, have reported health problems associated with contamination (De Farias 2023). Elevated levels of dissolved aluminum have been identified, particularly during the rainy season (Santos et al. 2023), along with significant increases in lipid peroxidation in fish from mining-affected areas (Cantanhêde et al. 2022). In Vila do Conde, parameters such as pH, total solids, and nitrate concentrations indicate periods during which water is unsuitable for human consumption (Medeiros et al. 2016). Lead levels up to nine times higher have been detected in residents living near the industrial zone compared to control populations (Queiroz et al. 2019), in addition to concerning cadmium concentrations in blood samples (Naka et al. 2020). Soil contamination by copper, nickel, and zinc has also been recorded in areas near the former landfill site (Matos et al. 2023).

In this context, understanding epigenetic responses, such as alterations in CpG island methylation that regulate gene transcription and essential biological processes is fundamental to elucidating the effects of contaminants on species in the region (Gardiner-Garden and Frommer 1987; Wutz et al. 1997; Darwall et al. 2018). Methylation of these regions results in the stable silencing of genes and plays a critical role in establishing epigenetic phenomena such as genomic imprinting (Wutz et al. 1997; Zwart et al. 2001). Moreover, CpG island methylation is essential for the maintenance of cellular identity, with the enzyme *Dnmt1*, responsible for preserving methylation patterns, being crucial to this process (Moore et al. 2012). The study of promoter methylation and its response to environmental factors is key to understanding the molecular mechanisms underlying pollutant-induced phenotypic changes, including those triggered by pesticides, metals, and other toxic compounds (Herman et al. 1996; Chen et al. 2018). Comprehending these epigenetic responses may provide complementary information for environmental monitoring, helping to elucidate how environmental disturbances influence biodiversity and species adaptation.

In this regard, it becomes increasingly relevant to integrate epigenetic studies with the analysis of genes directly involved in physiological responses to environmental contaminants. Among these, genes associated with xenobiotic metabolism are particularly notable (Johnson et al. 2012), especially those belonging to the *cyp* (cytochrome P450) and *slc* (solute carrier) families (Esteves et al. 2021). In aquatic environments, xenobiotics encompass pesticides, herbicides, polycyclic aromatic hydrocarbons (PAHs), metals, pharmaceuticals intended for human and veterinary use, synthetic hormones, food additives, and microplastics. These pollutants can accumulate within ecosystems and interfere with essential cellular processes (DeLorenzo et al. 2001; Croom 2012).

In the present study, the selection of target genes was also guided by an epigenetic criterion. Promoter regions of candidate genes from the CYP and SLC families were screened for the presence of CpG islands using MethPrimer software. Among the analyzed genes, *cyp1b1* and *slc16a12b* exhibited well-defined CpG-rich regions within their promoters, making them suitable candidates for investigating methylation-dependent regulation. While *slc16a12b* is primarily recognized for its role in the transport of organic compounds, members of the SLC family are broadly involved in the cellular redistribution and elimination of metabolites, including those derived from xenobiotic biotransformation. Therefore, evaluating both a Phase I metabolism gene *cyp1b1* and a transporter gene *slc16a12b* allows a more integrated assessment of detoxification-related processes under environmental exposure. The identification of CpG islands in promoter regions supports the hypothesis that these genes may be epigenetically regulated under environmental stress conditions.

The biotransformation of these compounds primarily occurs through three phases. In Phase I, CYP enzymes catalyze oxidation reactions, rendering molecules more hydrophilic and thus more easily excreted (Uno et al. 2012). In Phase II, these substances may be conjugated to polar groups, whereas Phase III transporters, such as those from the *slc* family, mediate their cellular elimination (Döring and Petzinger 2014). However, the efficiency of this detoxification system can be profoundly affected by environmental factors that modulate the expression and activity of these enzymes and transporters (Felmlee et al. 2020). In fish, this defense network against xenobiotics is particularly important, given the constant exposure of these organisms to the water column, sediments, and other compartments of aquatic ecosystems (Kaminsky 2006). Prolonged exposure to xenobiotics has been shown to compromise homeostasis and trigger toxic responses, including through the epigenetic dysregulation of genes associated with metabolism and cellular defense (Uno et al. 2012). Alterations in methylation levels at the promoters of CYP and SLC genes can modify their expression, thereby influencing the organisms’ ability to metabolize and eliminate toxic compounds (Kaminsky 2006; Romano et al. 2014).

In light of this context, the present study aims to investigate the expression patterns and methylation profiles of the *cyp1b1* and *slc16a12b* genes in *Geophagus surinamensis*, a species native to the Amazon Basin. These genes were selected due to their central roles in the biotransformation of xenobiotics (*cyp1b1*) and the transport of metabolites and organic compounds (*slc16a12b*), processes directly impacted by environmental contaminants. Analyzing methylation in the promoters of *cyp1b1* and *slc16a12b* allows for the inference of how epigenetic modifications may modulate gene expression, thereby affecting the efficiency of physiological responses to chemical stress. Investigating these mechanisms in native species is justified by the need to understand how Amazonian organisms respond to increasing anthropogenic pressures. In addition to advancing knowledge of the epigenetic mechanisms involved, this approach contributes to the development of sensitive and effective biomarkers for environmental monitoring. Such an endeavor is particularly relevant in megadiverse and ecologically sensitive ecosystems like the Amazon, where the cumulative effects of pollution threaten biodiversity, ecosystem services, and the food security of human populations dependent on these resources.

## Methods

### Study area

**Fig. 1:**
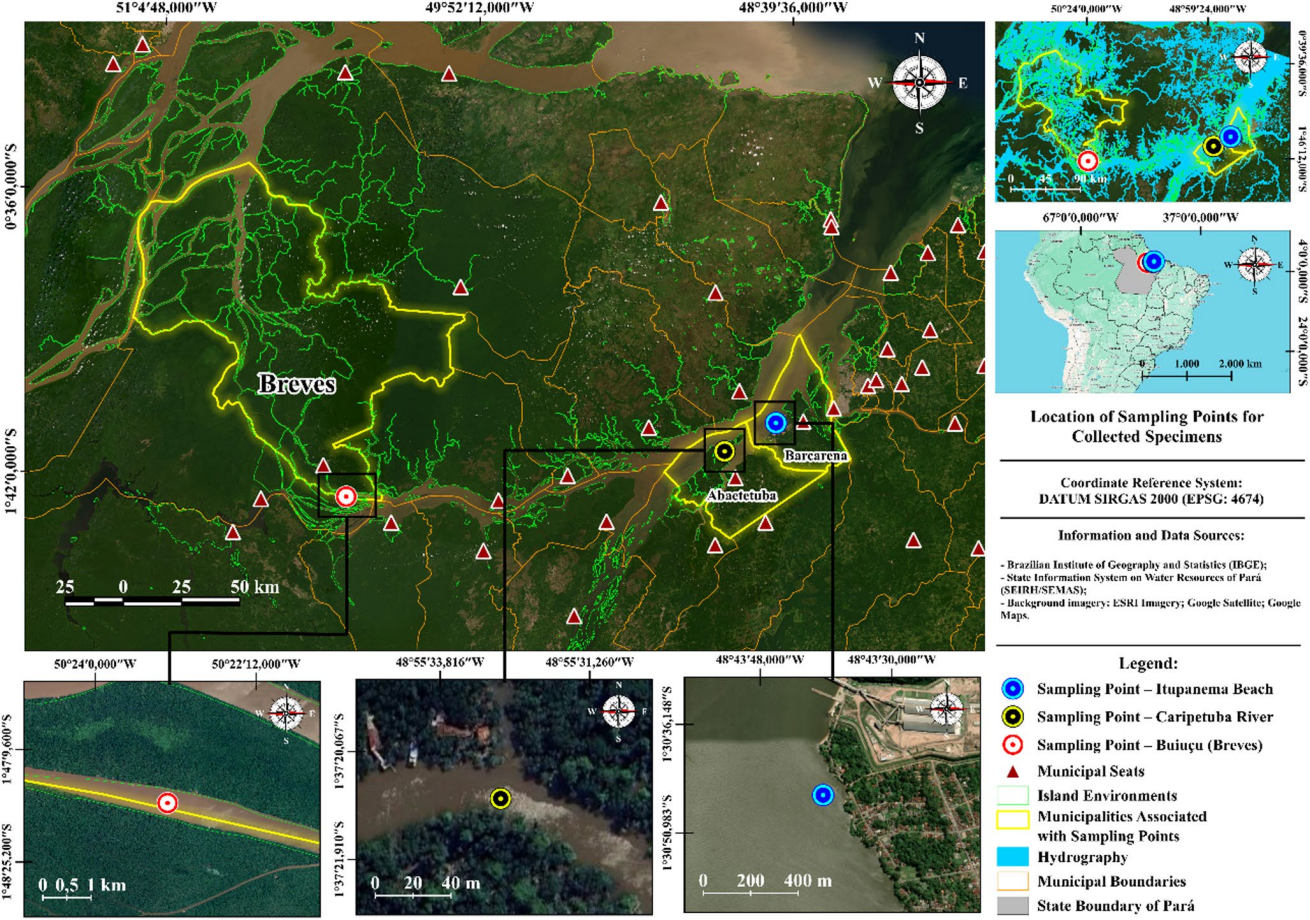
Map of the species sampling sites

The study was conducted using samples collected in the municipalities of Breves, Abaetetuba, and Barcarena, located in the state of Pará, Brazil. Breves has a tropical rainforest climate (Af, Köppen–Geiger), with a mean annual temperature of 26.8 °C and year-round high precipitation. The municipality covers 9,566 km² and is situated in the Marajó mesoregion. Barcarena presents a humid tropical rainforest climate (Af), with a mean annual temperature of 25.9 °C and a territorial area of 1,310 km², located in the Metropolitan mesoregion of Belém. Abaetetuba exhibits an equatorial monsoon climate (Am), with a mean annual temperature of 27 °C, and belongs to the Cametá microregion and the Northeastern Pará mesoregion, bordering Barcarena (Fig. 1). Territorial area data were obtained from the Brazilian Institute of Geography and Statistics (IBGE, 2022); Aguilar et al. (2017); Freitas et al. (2020) and Dias and Penner (2021).

The Barcarena Industrial Complex, located in Pará, Brazil, constitutes a significant study area (Fig. 1), as the municipality hosts major metallurgical, mining, and chemical enterprises (De Farias 2023). Episodes of industrial waste spills, such as bauxite and toxic substance leaks into the Murucupi and Pará rivers, underscore the region’s environmental vulnerability and its impacts on aquatic biodiversity and the health of riverine populations (Morales et al. 2025). The intensification of port and industrial activities has led to changes in water and soil quality, also affecting traditional communities. Consequently, Barcarena represents a hotspot for environmental research, particularly for investigating pollution-induced epigenetic alterations in aquatic organisms.

Although Breves cannot be considered a pristine environment due to the presence of urban settlements and potential domestic effluents typical of Amazonian riverine municipalities, it is geographically distant from large industrial complexes and mining activities. In contrast to Barcarena, which hosts a major metallurgical and industrial hub, Breves is characterized primarily by urban activities. Therefore, Breves was used as a comparative reference site representing lower industrial pressure rather than an absolutely uncontaminated control.

### Sample collection

Adult specimens of *Geophagus surinamensis* (Acará-tinga) were collected in 2021, during the months of March and April, at sampling sites located in the municipalities of Breves, Abaetetuba, and Barcarena, in the state of Pará, Brazil, under collection license SISBIO 21078-13/CEUA 8,803,211,223. Specimens were captured using artisanal longlines (espinhel), with each site subjected to a standardized sampling effort of 12 h during the nighttime period. Eight specimens were obtained from the Buiuçu channel (1°47’45.6"S 50°23’11.4"W) in the Breves region, ten specimens from the Caripetuba River (01°37′23.49″S 048°55′33″W) in Abaetetuba, and eight specimens from the vicinity of Itupanema Beach (1°30’45.8"S 48°43’39.4"W) in the municipality of Barcarena, totaling 26 specimens for experimental analyses. The sampled individuals presented a mean total length (TL) of 22.5 ± 2.5 cm and a mean body weight of 425 ± 100 g (mean ± SD).

### Biological material processing

Abdominal incisions were made in a caudo-cranial direction, from the genital opening to the gills, to obtain the target tissues (liver, muscle, and gills) from each specimen. The samples were immediately frozen in liquid nitrogen and stored in a freezer at − 80 °C. The selection of each tissue was based on its relevance to environmental degradation issues and their biological implications. The liver was chosen due to its central role in detoxification processes involving hepatocytes, through reactions such as acetylation, conjugation, methylation, and oxidation (Sciandrello et al. 2004). Muscle tissue was selected because the flesh of *Geophagus surinamensis* constitutes a dietary resource for human populations (Sousa et al. 2020) and, therefore, is of particular interest for public health considerations. Gills were included due to their susceptibility to functional alterations in gas exchange processes because of the accumulation of high concentrations of metals, given their continuous contact with the aquatic environment (Sakuragui et al. 2003; Weber et al. 2008). Species identification was performed based on external morphological characteristics in accordance with the taxonomic framework proposed by Kullander (1986, 1998) and current nomenclatural data from Eschmeyer’s Catalog of Fishes (Fricke et al. 2024).

### DNA and RNA extraction

DNA was extracted using a phenol: chloroform: isoamyl alcohol solution (25:24:1), following the manufacturer’s instructions. DNA quantification was performed using a BioTek microplate spectrophotometer, model EPOCH. RNA extraction was carried out using Trizol^®^ Reagent (Thermo Fisher Scientific), also following the manufacturer’s protocol. RNA integrity (RIN – RNA Integrity Number) was assessed with a 2011 Bioanalyzer (Agilent), and only samples with a RIN value greater than 7 were used in subsequent experiments.

### Evaluation of gene promoter profiles by MSP (Methylation-Specific PCR)

The Methylation-Specific PCR (MSP) technique, as described by Herman et al. (1996), allows for the rapid assessment of the methylation status at CpG sites within a CpG island. This technique is based on the conversion of unmethylated cytosines to uracils by sodium bisulfite treatment, while methylated cytosines remain unchanged. Subsequent amplification is performed using primers specific for methylated DNA (primer M) and unmethylated DNA (primer U). The methylation status is interpreted as follows: amplification with primer M only indicates methylation; amplification with primer U only indicates unmethylation; amplification with both primers indicates hemimethylation.

Promoter sequences were obtained from the available genomic data of *Oreochromis niloticus*. CpG island prediction and primer design were performed using the MethPrimer software. Species-specific primers were designed targeting bisulfite-converted DNA, avoiding CpG sites within the primer binding regions whenever possible to ensure specificity. The primer sequences are provided in the images (Supplementary Figure S2) in the supplementary file.

The DNA conversion process began with the addition of 2 µL of sodium hydroxide (3 M) to 2 µg of DNA in 20 µL, followed by incubation at 40 °C for 15 min. Subsequently, sodium bisulfite (2 M), urea (6.25 M), and hydroquinone (10mM) were added, and the mixture was incubated in a thermal cycler at 55 °C for 15 min and 95 °C for 30 s, repeated over 20 cycles. DNA purification was performed using the PureLink Genomic DNA Mini Kit (Invitrogen^®^), followed by ethanol precipitation and storage at − 20 °C.

The MSP amplification reaction was prepared in a final volume of 15 µL, consisting of 9.76 µL of ultrapure water, 0.56 µL of dNTPs (8mM), 0.45 µL of MgCl₂, 1.5 µL of Taq Polymerase buffer (10X), 0.4 µL of each MSP primer, 0.13 µL of Taq Polymerase (5U/µL), and 1 µL of converted genomic DNA (50 ng). PCR conditions included an initial denaturation at 95 °C for 5 min, followed by 35 cycles of 95 °C for 1 min, 60 °C for 30 s, and 72 °C for 30 s, with a final extension at 72 °C for 30 s, and storage at 4 °C. Results were analyzed on a 2% agarose gel.

### Gene expression analysis (RT-qPCR)

Liver, gill, and muscle tissues from eight specimens at each sampling site were used for the analysis. cDNA synthesis was performed using the High Capacity cDNA Reverse Transcription Kit, following the manufacturer’s specifications (Thermo Fisher Scientific). One microliter of cDNA (4 ng/µL) was amplified using the GoTaq^®^ qPCR Master Mix Kit (Promega) and 400 nM of each primer in a final reaction volume of 20 µL. Cycling conditions consisted of an initial denaturation at 95°C for 10 minutes, followed by 40 cycles of 95°C for 15 seconds and annealing/extension at 60°C (for *cyp1b1*) or 50°C (for *slc16a12b*) for 1 minute. Gene expression levels were detected using the StepOne Plus Real-Time PCR System (Thermo Fisher Scientific). The data were normalized using the Q-Gene software using the Ubiquitin-conjugating enzyme E gene (ubce) as a reference (Muller et al. 2002; Simon 2003).”

### Statistical analyses

Statistical analyses performed using GraphPad Prism software, version 10.4.1. Data normality, when necessary, assessed using the Shapiro-Wilk test. Homogeneity of variances was evaluated using Levene’s test. Parametric data involving three or more groups were analyzed by analysis of variance (ANOVA), followed by Tukey’s post hoc test, considering a significance level of 5% (α = 0.05). For correlation analyses, Pearson’s linear correlation test (r) applied. The strength of the correlation was interpreted as strong when r was close to 1 or − 1, and weak or nonexistent when r approached 0. Additionally, a two-tailed p-value test performed to determine the statistical significance of the observed correlations.

## Results

### Evaluation of gene promoter profiles by MSP

**Fig. 2:**
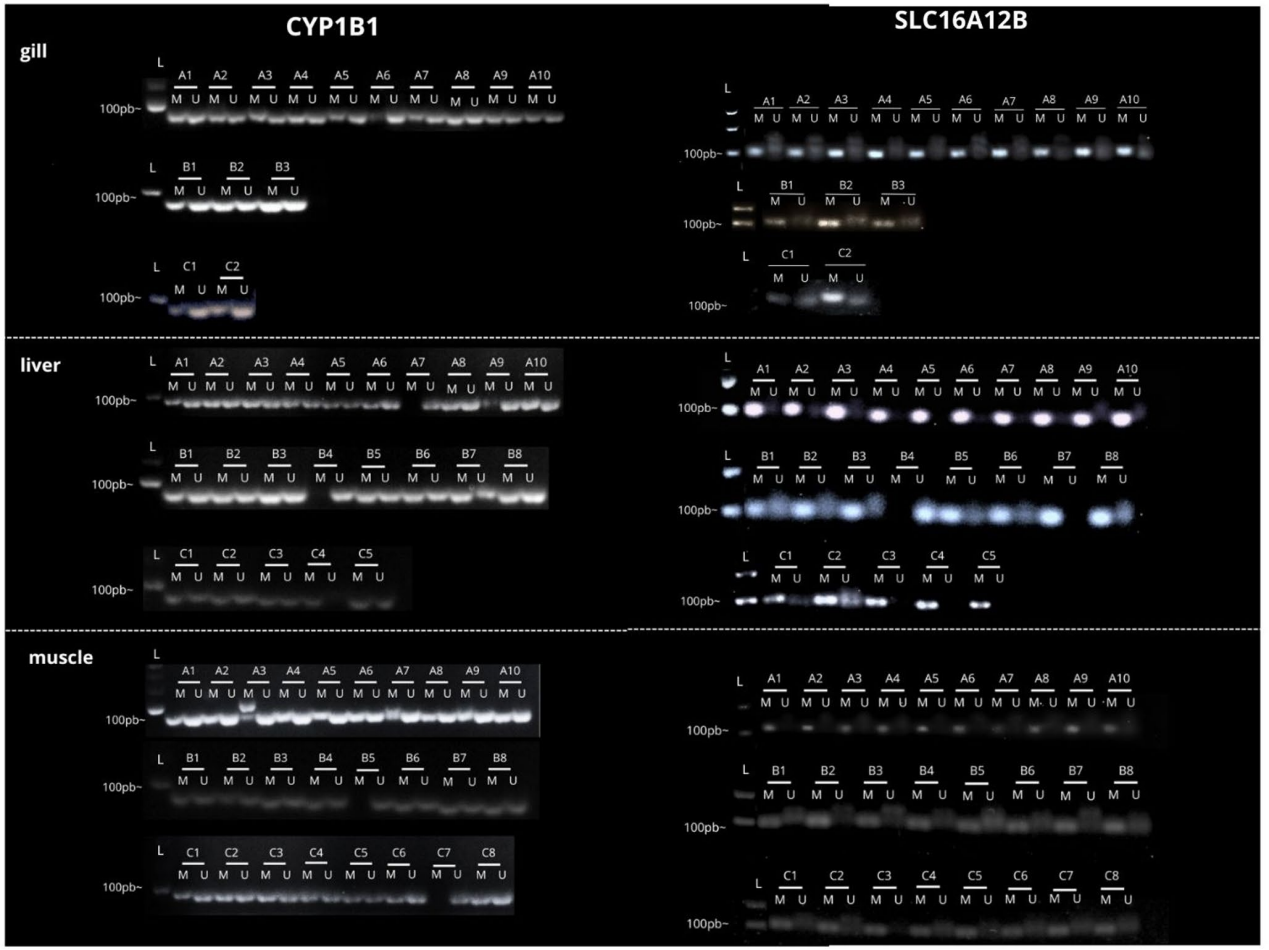
Methylation-specific PCR (MSP) analysis of *cyp1b1* and *slc16a12b* promoter regions, visualized on a 2% agarose gel. Amplification products for methylated (M) and unmethylated (U) DNA are shown for gill, liver, and muscle tissues. Individuals are identified by number and sampling locality: Abaetetuba (A), Barcarena (B), and Breves (C). Molecular weight markers (L) are indicated for size reference

The hemimethylation pattern was observed across all studied tissues and sampling sites for the *cyp1b1* gene, whereas methylation patterns were more frequently detected for *slc16a12b *(Fig. 2). For gel analysis, only well-defined amplification bands were considered, excluding smear patterns. Among the analyzed tissues, the liver exhibited the greatest variability; however, hemimethylation remained predominant for *cyp1b1*.

In gill tissue, analysis of the promoter region of *cyp1b1* revealed a consistent hemimethylation pattern in all individuals, while *slc16a12b* displayed a uniform methylation pattern throughout the sampled populations (Fig. 2). A similar pattern was observed in muscle tissue, with the exception of one individual from the Barcarena population (B5) and one from the Breves population (C7), which exhibited a non-methylated pattern in the *cyp1b1* promoter region (Fig. 2).

Greater discrepancies in methylation patterns were detected in liver tissue. In the Abaetetuba population, two individuals (A7 and A9) showed a non-methylated pattern for *cyp1b1*, whereas the methylation status of *slc16a12b* was maintained in all analyzed individuals (Fig. 2). In the Barcarena population, non-methylation was observed for both genes. In contrast, in the Breves population, one individual (C4) exhibited a methylated pattern in the *cyp1b1* promoter region, while the *slc16a12b* promoter remained methylated in all individuals (Fig. 2).

Not all target tissues (muscle, liver, and gills) could be obtained from every individual. Sample preservation conditions, transportation time to the laboratories, and logistical constraints associated with sampling in natural environments affected tissue availability. Although the absence of some tissues from Barcarena and Breves limited the overall scope of the study, the analysis of liver and gill tissues still enabled the assessment of methylation alterations in the investigated tissues.

### Expression of *cyp1b1* and *slc16a12b* genes

#### Overall expression of cyp1b1 and slc16a12b genes

**Fig. 3:**
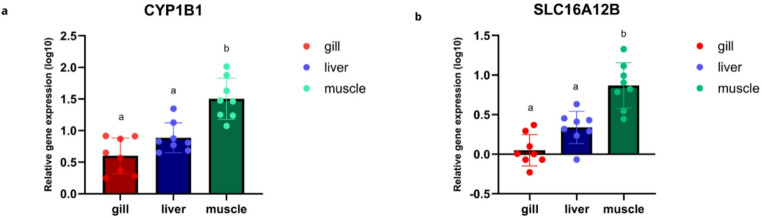
(**a**) Bar graph representing the overall expression of the *cyp1b1* gene in gill, liver, and muscle tissues of *Geophagus surinamensis*. (**b**) Bar graph representing the overall expression of the *slc16a12b* gene in gill, liver, and muscle tissues of *Geophagus surinamensis*, based on RT-qPCR analysis. Different letters indicate statistically significant differences between groups. The p-values were determined by one-way ANOVA followed by Tukey’s post hoc test, with a significance level of 5% (α = 0.05)

The results demonstrate that muscle tissue exhibited the highest expression levels for both genes, followed by liver and gill tissues, respectively. Statistical analysis indicated a significant difference in gene expression between muscle tissue and the gill and liver tissues (*p* < 0.05). *cyp1b1* expression reached a maximum value of 2.5 (log₁₀), whereas *slc16a12b* expression reached a maximum value of 1.5 (log₁₀) (Fig. 3).

#### cyp1b1

**Fig. 4:**
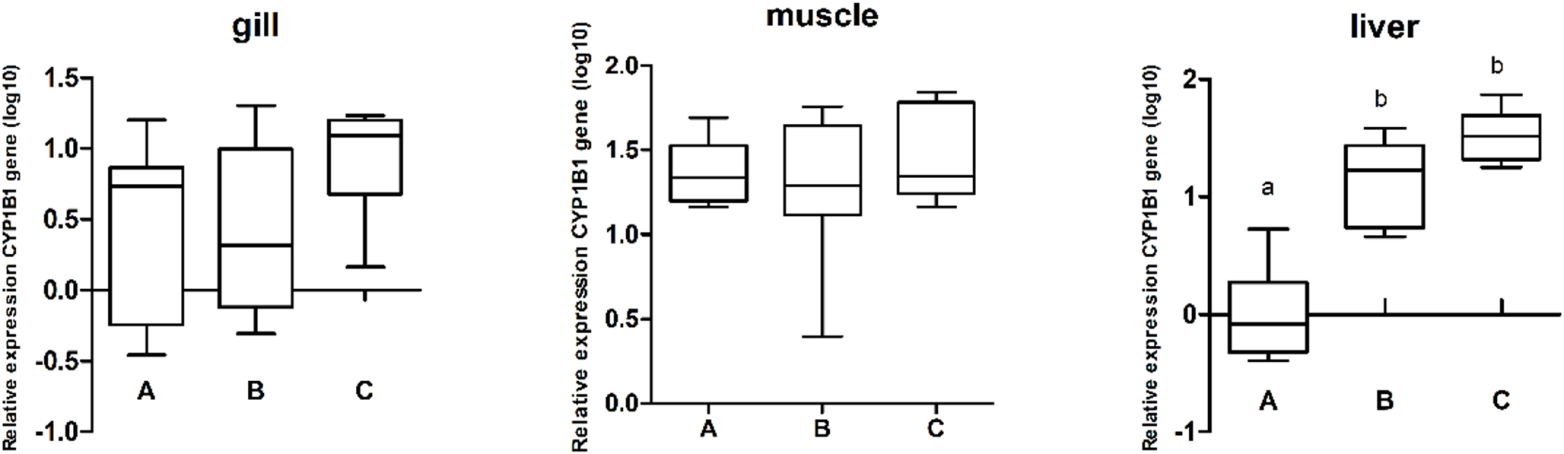
Bar graphs representing *cyp1b1* gene expression in gill, muscle and liver tissues of *Geophagus surinamensis* collected from Abaetetuba (A), Barcarena (B), and Breves (C). Different letters indicate statistically significant differences between groups. The p-values were determined by one-way ANOVA followed by Tukey’s post hoc test, with a significance level of 5% (α = 0.05)

The expression levels of the *cyp1b1* gene were evaluated in the gill, liver, and muscle tissues of *Geophagus surinamensis* collected from Breves, Abaetetuba, and Barcarena (Fig. 3a). In gill and muscle tissues, no statistically significant differences in *cyp1b1* expression levels were observed among the sampling groups, although there was a tendency toward lower *cyp1b1* expression in the muscle tissue of Abaetetuba compared to Breves and Barcarena. In liver tissue, *cyp1b1* expression was significantly lower in the Abaetetuba samples compared to Breves and Barcarena (*p* < 0.0001; α = 0.05) (Fig. 4). Heatmap analysis revealed a trend of higher *cyp1b1* expression in gill tissues from Breves (C) compared to Abaetetuba (A) and Barcarena (B), although these differences were not statistically significant (Supplementary Figure S1). The statistical differences identified in liver tissue were particularly pronounced in the heatmap analysis of hepatic samples.

#### slc16a12b

**Fig. 5:**
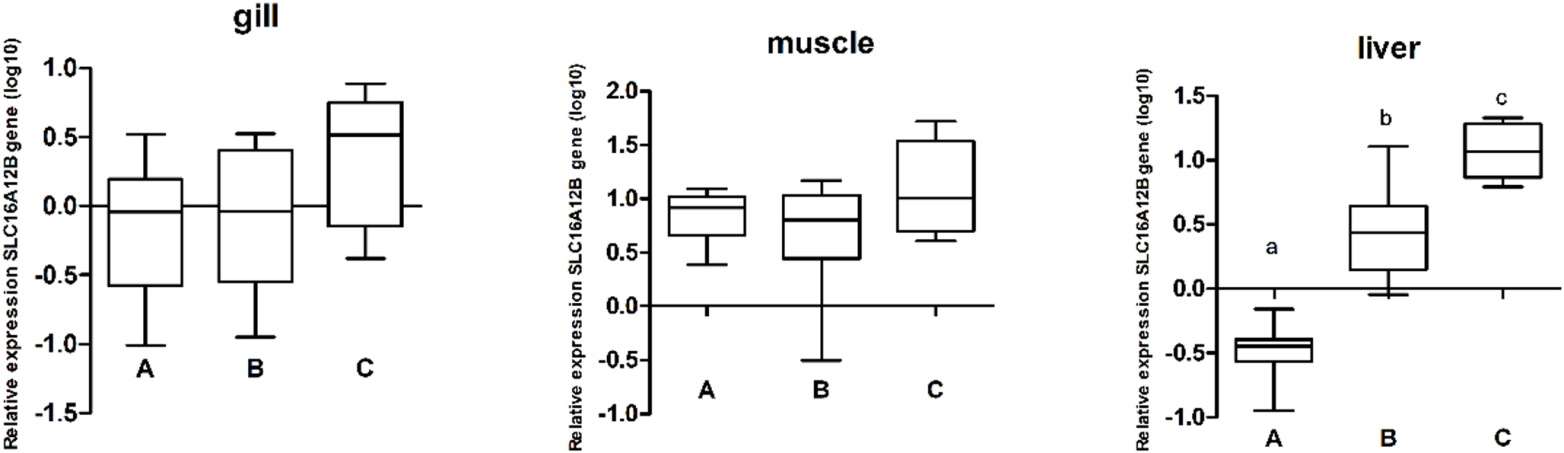
Bar graphs representing *slc16a12b* gene expression in gill, muscle and liver tissues of *Geophagus surinamensis* collected from Abaetetuba (A), Barcarena (B), and Breves (C). Different letters indicate statistically significant differences between groups. The p-values were determined by one-way ANOVA followed by Tukey’s post hoc test, with a significance level of 5% (α = 0.05)

The expression levels of the *slc16a12b* gene were evaluated in the gill, liver, and muscle tissues of *Geophagus surinamensis* collected from Breves, Abaetetuba, and Barcarena (Fig. 3b). In gill and muscle tissues, no statistically significant differences in gene expression levels were observed among the sampling groups, although there was a tendency toward higher expression in both tissues from Breves compared to Abaetetuba and Barcarena. In liver tissue, significant differences were observed among the three sampling sites, represented by the letters a, b, and c (*p* < 0.0004; α = 0.05) (Fig. 5). Heatmap analysis further highlighted these statistical differences, with color variation clearly differentiating liver tissue expression patterns (Supplementary Figure S1).

#### Correlation of cyp1b1 and slc16a12b gene expression

**Fig. 6:**
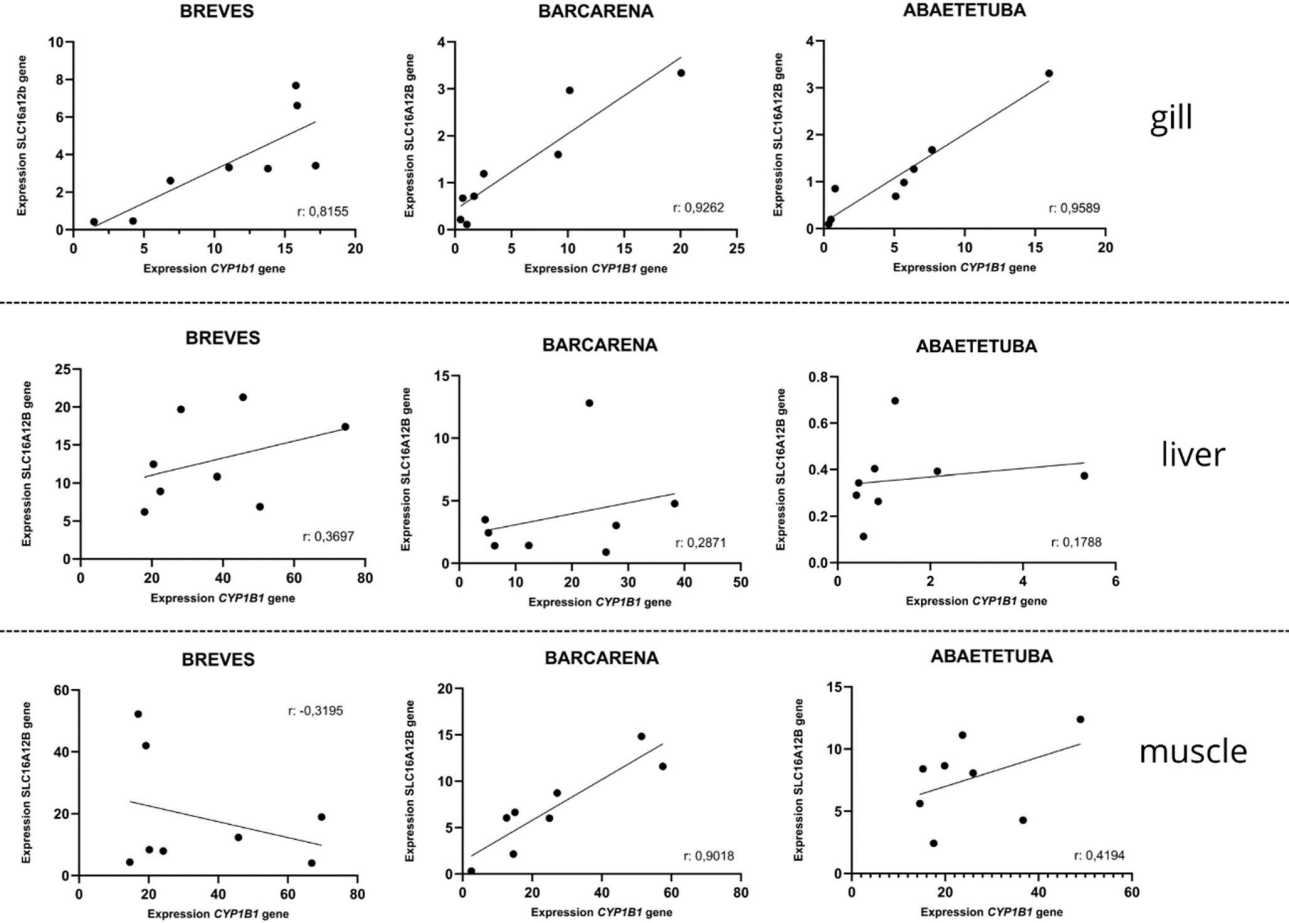
Linear correlation plots between *cyp1b1* and slc16a12b gene expression levels in gill, liver, and muscle tissues of *Geophagus surinamensis* collected from Abaetetuba, Barcarena, and Breves. Pearson’s correlation coefficient (r), ranging from − 1 to + 1, was used for the analysis. Values near + 1 indicate a strong positive correlation, values near − 1 indicate a strong negative correlation, and values near 0 indicate the absence of a linear correlation

Positive and strong correlations between *cyp1b1* and *slc16a12b* gene expression levels were observed in gill tissues across all sampling sites: Abaetetuba (*r* = 0.9589, *p* = 0.0002), Barcarena (*r* = 0.9262, *p* = 0.0009), and Breves (*r* = 0.8155, *p* = 0.0136). In liver tissue, no significant correlation between the expression of the two genes was observed at any sampling site. In muscle tissue, a positive and strong correlation was detected in Barcarena (*r* = 0.9018, *p* = 0.0022) (Fig. 6).

## Discussion

The Barcarena Industrial Complex in Pará, Brazil, represents a major industrial hub whose expansion has resulted in significant environmental impacts, particularly metal contamination by aluminum, mercury, cadmium, and lead, compromising water quality, aquatic ecosystems, and human health (Lemos and Pimentel 2020; Coutinho et al. 2023; De Farias 2023). Elevated metal concentrations, frequently exceeding regulatory limits, especially during the rainy season, have been reported in water, soil, fish, and local populations, increasing toxic exposure and affecting food security in surrounding communities (Medeiros et al. 2016; Naka et al. 2020; Queiroz et al. 2019; Cantanhêde et al. 2022; Matos et al. 2023; Santos et al. 2023). Heavy metals and other contaminants are recognized modulators of DNA methylation across taxa, influencing transcriptional regulation and stress responses (Choi et al. 2008; Ho et al. 2012; Mauro et al. 2016; Ding et al. 2019; Montes-Castro et al. 2019; Bian and Gao 2021). DNA methylation in promoter regions is typically associated with transcriptional repression due to chromatin compaction and reduced accessibility of transcriptional machinery, whereas hypomethylation is generally linked to increased gene expression. In Amazonian systems, the additional input of pharmaceuticals, untreated sewage, and domestic waste further intensifies xenobiotic exposure, reinforcing the role of pollution in modulating epigenetic regulation in aquatic organisms (Zhou et al. 2001; Aniagu et al. 2008; Stromqvist et al. 2010; Ruiz-Hernandez et al. 2015; Medeiros et al. 2017; Sun et al. 2021). In the present study, a recurrent hemimethylation pattern in *cyp1b1* was observed across tissues and sampling sites (Fig. 2), suggesting a gene-specific epigenetic configuration within the analyzed environmental context. In contrast, *slc16a12b* showed a higher frequency of fully methylated patterns (Fig. 2), indicating distinct regulatory dynamics between genes involved in xenobiotic metabolism and membrane transport. The liver exhibited the greatest variability in methylation, although hemimethylation predominated for *cyp1b1*. Importantly, MSP provides qualitative rather than quantitative information; therefore, a direct correlation between methylation status and expression levels cannot be established. Although hemimethylation has been associated with epigenetic plasticity and adaptive transcriptional responses (Baccarelli and Bollati 2009), quantitative methylation approaches would be required to clarify its relationship with transcriptional activity. These findings align with global methylation data reported by Marcelino et al. (2025), indicating that tissue-specific global changes may coexist with gene-level modulation, particularly in genes central to xenobiotic metabolism. Muscle tissue exhibited the highest expression levels of both genes, followed by liver and gill, with significant differences between muscle and the other tissues (*p* < 0.05) (Fig. 3). Overall, *cyp1b1* expression exceeded that of slc16a12b, consistent with its established role in Phase I xenobiotic monooxygenation (Uno et al. 2012). The reduced hepatic expression of *cyp1b1* in Abaetetuba may reflect differences in contaminant load among sites. Metals and polycyclic aromatic hydrocarbons can either induce or inhibit CYP activity depending on exposure context (Kaminsky 2006; Ogunwole et al. 2021), potentially impairing biotransformation capacity. Experimental evidence demonstrates that heavy metals influence global DNA methylation in fish liver (Zhou et al. 2001), supporting the possibility of site-specific epigenetic modulation. Consistent with this, antioxidant studies indicate that the liver is particularly responsive to chemical stress due to its central metabolic role (Marcelino et al. 2025). Lower lipid peroxidation levels previously observed in Abaetetuba (Marcelino et al. 2025) may correspond to reduced activation of detoxification pathways, aligning with decreased hepatic *cyp1b1* expression. Together, these data suggest that local differences in contaminant exposure may modulate both oxidative and transcriptional responses. The higher hepatic expression of *slc16a12b*, with site-specific differences, supports its role in metabolite transport following xenobiotic biotransformation, consistent with the broader function of SLC family members in cellular transport and metabolic regulation (Felmlee et al. 2020). In gills, the trend toward increased *cyp1b1* and *slc16a12b* expression in Breves may reflect adaptive responses to dissolved contaminants, given the direct exposure of this tissue to the aquatic environment (Sakuragui et al. 2003; Weber et al. 2008). Although not always statistically significant, coordinated expression patterns in gill tissue suggest integrated detoxification and transport mechanisms under environmental pressure.

Correlation analyses further highlighted tissue-specific regulatory dynamics. In gills, strong positive correlations between *cyp1b1* and *slc16a12b* were observed across all sites (Fig. 6), suggesting coordinated xenobiotic metabolism and transport. Given that metals often accumulate in gill tissues (Zarazúa et al. 2014), this pattern may reflect direct environmental influence on this interface organ. In contrast, no significant correlations were detected in liver, possibly reflecting more complex regulatory networks involving additional transport pathways such as ABC transporters (Ferreira et al. 2014). In muscle, a significant positive correlation was observed only in Barcarena, potentially associated with bioaccumulation processes. Previous reports of mercury biomagnification in Amazonian fish (Souza-Araujo et al. 2016) and documented contamination events in the region (Morales et al. 2025; Bordalo et al. 2012) support the interpretation that local pollution may influence muscle gene regulation.

Despite evidence of biochemical alterations in previous studies (Cantanhêde et al. 2022; Marcelino et al. 2025), genetic investigations of these specific pathways in this species had not been reported. The present findings therefore provide novel insight into transcriptional and epigenetic modulation associated with environmental exposure.

Given the ecological and toxicological relevance of *cyp1b1* and *slc16a12b*, further investigations integrating molecular, histological, and physiological analyses would enhance understanding of contaminant impacts in Amazonian freshwater systems. Characterizing tissue lesions alongside gene expression could clarify functional consequences of pollutant exposure and strengthen biomarker development.

It is important to acknowledge that all specimens were collected during the rainy season, representing a temporal limitation. Seasonal hydrological variation in Amazonian rivers influences contaminant dynamics and biological responses, potentially affecting both gene expression and methylation patterns. Future studies incorporating multiple hydrological periods are necessary to determine the consistency of the observed molecular patterns throughout the annual cycle.

## Conclusion

This study revealed the influence of environmental contamination from the Barcarena Industrial Complex on aquatic organisms, with a focus on epigenetic effects in *Geophagus surinamensis*. The contrasting methylation patterns observed between the two genes suggest distinct epigenetic regulatory mechanisms. The consistent hemimethylation of *cyp1b1* across all studied tissues and collection sites may reflect an active or transitional regulatory state, potentially associated with dynamic responses to environmental stimuli. In contrast, the widespread methylation of *slc16a12b* may indicate a more stable silencing profile or a gene-specific epigenetic signature. These findings highlight that environmental or endogenous factors may differentially influence methylation status depending on gene function, tissue type, or local context, emphasizing the importance of gene-specific epigenetic monitoring in environmental and toxicological studies.

Moreover, the differential expression of *cyp1b1* and *slc16a12b* among the analyzed tissues reinforced the importance of epigenetic regulation in modulating biochemical responses to xenobiotics, highlighting the complexity of defense mechanisms employed by organisms against pollution.

This study is relevant to both the fields of epigenetics and environmental conservation in the amazon. By expanding the understanding of DNA methylation as a response to contaminants, the findings corroborate previous research linking pollution to epigenetic modifications across various species. Additionally, the observed epigenetic changes may serve as indicators of chronic pollutant exposure, suggesting their potential use as biomarkers for environmental monitoring. This aspect is critical for enhancing strategies to assess environmental impacts and for informing effective public policies aimed at mitigating the damages caused by industrial activities.

In the realm of conservation, the study underscores the urgency of implementing rigorous actions to contain the effects of industrialization on aquatic ecosystems and the human populations dependent on these resources. Heavy metal and other pollutant contamination threatens local biodiversity, disrupts natural habitats, and alters ecological dynamics. Furthermore, it poses significant health risks to riverine communities that rely on water resources for their livelihoods. Thus, it is essential to efficiently implement preventive and corrective measures, involving collaboration among the public sector, civil society, and industry.

Although this study has made a significant contribution, several gaps remain to be explored, such as the specificity of epigenetic mechanisms in response to different contaminants and the persistence of these modifications across generations. Investigations into the reversibility of epigenetic changes and the impact of pollution on gene regulation in bioindicator species are also necessary. The integration of genomic, transcriptomic, histological, and biochemical analyses could provide a more comprehensive understanding of the effects of pollution.

Finally, the expansion of environmental and epidemiological monitoring in the region is recommended to evaluate the long-term impacts of pollution and to support public policies aimed at ecosystem preservation and the protection of affected populations. Strengthening interdisciplinary research networks and promoting community engagement in environmental monitoring are essential for ensuring sustainable development, minimizing environmental damage, and improving the quality of life for local communities. The preservation of the Amazon’s natural resources for future generations will depend on continuous and integrated efforts.

## Supplementary information

Below is the link to the electronic supplementary material.

Supplementary Material 1

## Data Availability

The datasets generated and analyzed during the current study are available from the corresponding author on reasonable request **.**.
