# Supplementary material for: Effects of anthropogenic influences in the DNA methylation and expression of genes involved in the metabolism of Geophagus surinamensis from the Pará River (Amazon, Brazil)
Source: Ecotoxicology. 2026 Jun 1;35(5):110. doi: 10.1007/s10646-026-03093-w (PMC13226442; doi:10.1007/s10646-026-03093-w)

**Supplementary Material**

**Effects of Anthropogenic Influences in the DNA Methylation and Expression of Genes Involved in the Metabolism of *Geophagus surinamensis* from the Pará River (Amazon, Brazil)**

Luana Beatriz Sales Pinon^a^, Flávia dos Santos Tavares^a^, Luis Adriano Santos do Nascimento^b^, Bruno Rafael Ribeiro de Almeida^c^ Cesar Martins^d^, Adauto Lima Cardoso^d^, Renata Coelho Rodrigues Noronha^a,†^

^a^Laboratory of Genetics and Cell Biology, Center for Advanced Biodiversity Studies, Institute of Biological Sciences, Federal University of Pará, Belém, PA, Brazil, 66075-750.

^b^ Oils Laboratory, Institute of Biological Sciences, Federal University of Pará, Belém, PA, Brazil, 66075-750.

**^c^** State University of Pará (UEPA), Cametá, PA, Brazil, 68400-000.

**^d^**Integrative Genomics Laboratory, Institute of Biosciences, São Paulo State University, Botucatu, SP, Brazil, 18610-307.

^†^Correspondence author: RCRN -rcrn@ufpa.br

**Supplementary Figure S1.** Heatmaps showing individual gene expression profiles across different tissues. Panels **A–C** represent the relative expression of the ***slc16a12b*** gene in gill (A), liver (B), and muscle (C). Panels **D–F** represent the relative expression of the ***cyp1b1*** gene in gill (D), liver (E), and muscle (F). Rows correspond to individuals (1–8), and columns represent the sampling locations: **A – Abaetetuba**, **B – Barcarena**, and **C – Breves**. Color gradients indicate expression levels, with lighter colors corresponding to higher expression and darker colors to lower expression.


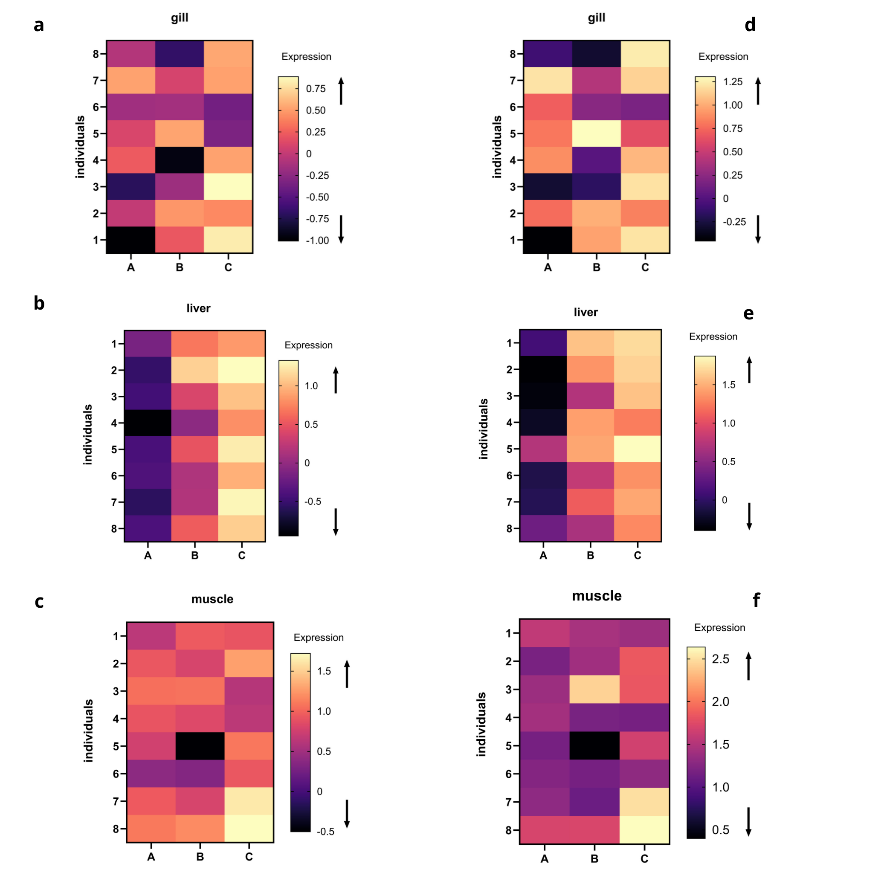


**Supplementary Figure S2.** Analysis of the promoter region for the identification of CpG islands using the **MethPrimer** software. Panel **A** corresponds to the search performed for the ***cyp1b1*** gene, and panel **B** corresponds to the search performed for the ***slc16a12b*** gene. The figure shows the GC content profile along the sequence, the distribution of CpG sites, and the prediction of potential candidate regions for designing specific primers for PCR after bisulfite treatment.


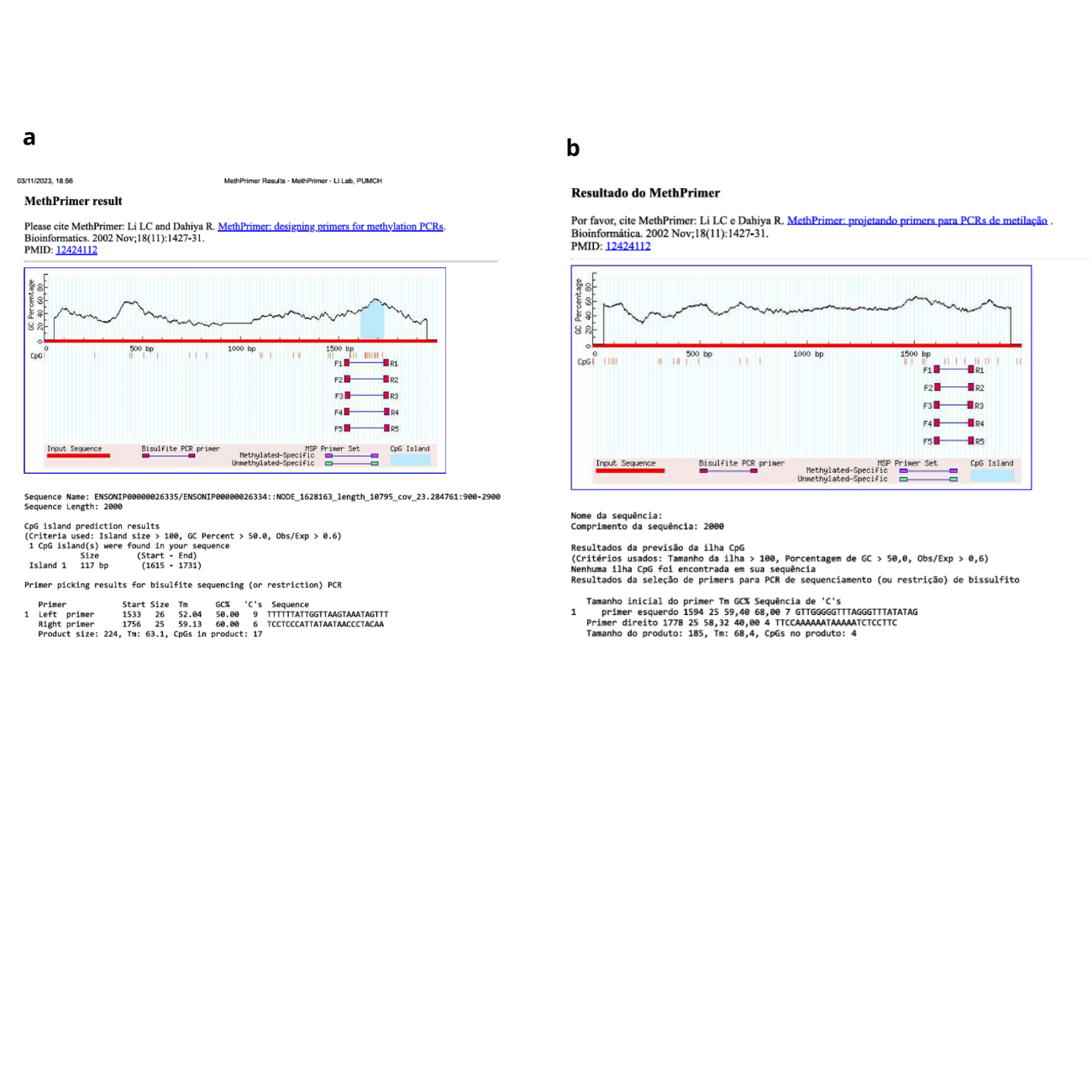

Supplement: Supplementary file 1 — Supplementary Material 1 [file 10646_2026_3093_MOESM1_ESM.docx]
